# Low dose of continuous – wave microwave irradiation did not cause temperature increase in muscles tissue adjacent to titanium alloy implants – an animal study

**DOI:** 10.1186/1471-2474-14-364

**Published:** 2013-12-23

**Authors:** Dongmei Ye, Yiming Xu, Tengfei Fu, Han Zhang, Xianxuan Feng, Gang Wang, Lan Jiang, Yuehong Bai

**Affiliations:** 1 Department of Rehabilitation, Shanghai Jiao Tong University Affiliated Sixth People's Hospital, Shanghai 200233, China

**Keywords:** Microwave, Titanium alloy, Implant, Temperature

## Abstract

**Background:**

Research studies on the influence of radiofrequency electromagnetic radiation on implants in vitro have failed to investigate temperature changes in the tissues adjacent to the implants under microwave therapy. We therefore, used a rabbit model in an effort to determine the impact of microwave therapy on temperature changes in tissues adjacent to the titanium alloy implants and the safety profile thereof.

**Methods:**

Titanium alloy internal fixation plates were implanted in New Zealand rabbits in the middle of femur. Microwave therapy was performed by a 2450 MHz microwave generator 3 days after the surgery. Temperature changes of muscles adjacent to the implants were recorded under exposure to dose-gradient microwave radiation from 20w to 60w.

**Results:**

Significant difference between control and microwave treatment group at peak temperatures (T_peak_) and temperature gap (T_gap=_ T_peak_-T_vally_) were observed in deep muscles (T_peak_, 41.63 ± 0.21°C vs. 44.40 ± 0.17°C, *P* < 0.01; T_gap_, 5.33 ± 0.21°C vs. 8.10 ± 0.36°C, *P* < 0.01) and superficial muscles (T_peak_, 41.53 ± 0.15°C vs. 42.03 ± 0.23°C, *P* = 0.04; T_gap_, 5.23 ± 0.21°C vs. 5.80 ± 0.17°C, *P* = 0.013) under 60 w, and deep muscles (T_peak_, 40.93 ± 0.25°C vs. 41.87 ± 0.23°C, *P* = 0.01; T_gap_, 4.73 ± 0.20°C vs. 5.63 ± 0.35°C, *P* = 0.037) under 50w, but not under 20, 30 and 40w.

**Conclusion:**

Our results suggest that low-dose (20w-40w) continuous-wave microwave irradiation delivered by a 2450 MHz microwave generator might be a promising treatment for patients with titanium alloy internal fixation, as it did not raise temperature in muscle tissues adjacent to the titanium alloy implant.

## Background

Microwave radiation is defined as that with a frequency of 300 MHz-300GHz, which lies on the electromagnetic spectrum between radiofrequency (RF) and infrared radiation. In general, microwaves could be focused to produce physiological heating in deep body, for example, increased temperature over 40°C [1], increased blood flow [2,3], decreased pain [4], and alterations in the physical properties of fibrous tissues [5,6]. The clinical application of microwaves improved range of motion in joints, and accelerated the resolution of hematoma and fracture healing [7]. Microwave treatment is also used in the cure and rehabilitation of injuries and chronic inflammation of bone [6,8-12], joint [13,14], muscle and tendon [15,16]. Frequencies historically used in the rehabilitation were 2450[17], 915 [6,18-22], 434 (with surface cooling) [6,20] and 27.12 MHz [23]. The efficacy of an electromagnetic wave device depends on the target site and temperature.

Surgically implanted metal plates, screws, and pins in the treatment field are an absolute contraindication for microwave application [8]. It is thought that electromagnetic radiation generated by microwave diathermy can be reflected, refracted or transmitted at the interface between tissues and metal implants [8,24], resulting in rapid elevation in temperature and tissue damage locally [25-29]. However, some doctors believed that this contraindication appeared to be based on “common sense” and consensus rather than evidence-based practice, in vivo. Titanium implants are widely used clinically since titanium ion is non-magnetic [30,31]. It has a lower magnetic permeability and electric conductivity than common magnetic materials, and consequently less absorption of radio frequency (RF) [32]. The findings from studies in vitro indicated that the temperature changes of titanium and titanium alloys in RF electromagnetic field were lower compared with other metallic implants [27,33]. In fact, the safety of shortwave diathermy has been proven in clinical case reports. Seiger [34] applied pulsed shortwave diathermy (27.12 MHz, 800 pps, 400 microseconds (48 W), 20 min) over a patient’s ankle with orthopedic metal implants, resulting in improved ankle range of motion (ROM). No complaints of discomfort, pain, or burning sensation occurred. Another report similarly suggested no negative effects during the short-term follow-up [35]. However, muscle temperature in the thigh was not monitored during the treatment. Although the patients’ complaints determined the temperature increase to some degree, a huge difference existed between thermal pain threshold and hyperthermia-induced injury [6,36]. As temperature measurement entailed an invasive approach, we designed an animal experiment to observe the transient temperature changes adjacent to the implants. Additionally, previous research involving RF electromagnetic field derived from mobile phones rarely indicated any risk associated with implants, though frequencies of 900[29], 1800 [28] and 2450 MHz [37,38] enhanced specific absorption rates (SAR). Therefore, the current study was based on the assumption that no dramatic temperature increases occurred in tissues around titanium alloy implants with a common therapeutic dose of microwave radiation (20-60w) used in rehabilitation.

Evidence supporting temperature changes in the tissues adjacent to the implants under microwave therapy is limited. In the current research, we modeled the worst possible cases of the most common implants under microwave irradiation in the rehabilitation of bone and muscle injuries. We implanted a titanium alloy into the femur of rabbit. We employed the highest frequency of 2450 MHz microwave generators at a treatment dose gradient of 20 w to 60 w, which are clinically used in rehabilitation. Temperature changes of muscles adjacent to the implants were recorded during the microwave exposure. Our objective was to determine the influence of microwave exposure on the temperature in tissues adjacent to a titanium alloy and the safety profile of microwave therapy.

## Methods

### Ethics statement

All the experimental procedures involving animals were conducted under a protocol reviewed and approved by the Animal Welfare and Ethics Committee of Shanghai Sixth People’s Hospital (Permit Number: SYXK(Hu) 2011–0128). All animal work was carried out in accordance with national and international guidelines to minimize suffering to animals.

### Animal model instruction

Eight New Zealand white rabbits aged 16–18 weeks and weighing 2.0 to 2.5 kg were used in this study. All rabbits were randomly divided into the treatment group (n = 5) and the control group (n = 3). The rabbits were anesthetized with an intravenous injection of pentobarbital sodium (30 mg/kg). The femoral upper end was exposed on the outside of the right upper thigh incision. The internal fixation plate was composed of a titanium alloy plate (4.60 ± 0.24 × 0.42 ± 0.08 cm) with seven holes and screws (LCP System, Synthes Company, USA), which were planted on right femoral upper end of the experimental group. Control animals underwent the same surgical procedure without titanium alloy implants. All rabbits were injected intramuscularly with penicillin (800,000 units) 3 days post-operation.

### Microwave diathermy

Three days after operation, microwave therapy was administered to both the treatment group and the control group. The rabbits were anesthetized with an intravenous injection of pentobarbital sodium (30 mg/kg). Treatment regimen consisted of microwave diathermy to the right upper thigh using the microwave diathermy system (PM-800, ITO Company, Japan). Microwave generator operated at a frequency of 2450 MHz. Continuous-wave microwave radiation was administered at a treatment dose gradient of 20 w, 30 w, 40 w, 50 w and 60 w, respectively, in the same regimen for 15 minutes. Changes in muscle temperature were recorded during microwave exposure. Each dose of microwave treatment was applied at intervals of 0.5 hour.

### Temperature measurements

Anti-interference couple thermometer (FHC, ME-04008, BOWDOINHAM, USA) was used for temperature measurement during diathermy. The sensor was inserted at different muscular depths over the titanium alloy plate, including deep muscles (5 mm above the hole) and superficial muscles (30 mm above the hole). Temperatures were recorded perpendicular to the middle hole of the titanium alloy plate, which was verified in a preliminary study. The temperatures of control group were tested similarly. The temperature of the laboratory was maintained at 24°C. Temperatures were recorded at the rate of 1 per minute for 15 minutes. We allowed 30 minutes for the rabbits to return to normal body temperature after each diathermy.

### Statistic

All the results were presented as the mean ± SD. Students’ unpaired t-test and one-way analysis of variance were performed with the SPSS 19.0 software. Statistical significance was set at *P* < 0.05.

## Results

### Preliminary study

**Figure 1:**
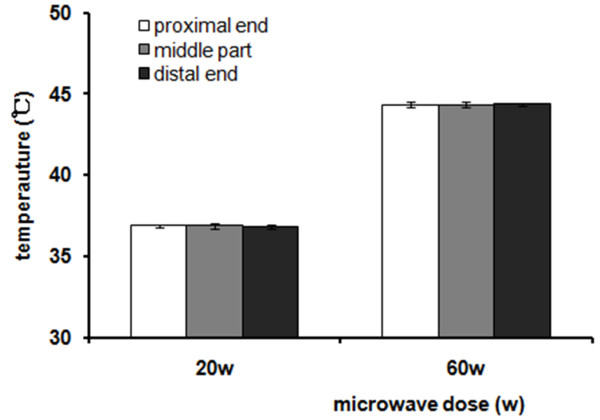
**Temperature peaks in an implant.** No significant difference in temperature peaks was observed with the three positions of the implant (all *P* > 0.05).

Previous studies observed that the electromagnetic field lines were focused on an edge or toward an end of implant leading to a high local flux [28,39]. Hence we performed the preliminary study to determine the position of insertion of the sensor. We measured the temperature changes at both ends and in the middle of an implant under 20w and 60w microwave exposure, in vivo. Temporal temperatures at 5 min, 10 min, and 15 min were recorded, respectively. The peak temperatures appeared toward the end of treatment. No significant differences in temperature changes were observed among the three positions at each time point (all *P* > 0.05) (T_peak_ shown in Figure 1). Since the microwave irradiation focused on the middle of the implants, the two measuring positions of temperature were perpendicular to the middle hole of the titanium alloy plate.

### Temporal temperature changes in deep muscles

**Figure 2:**
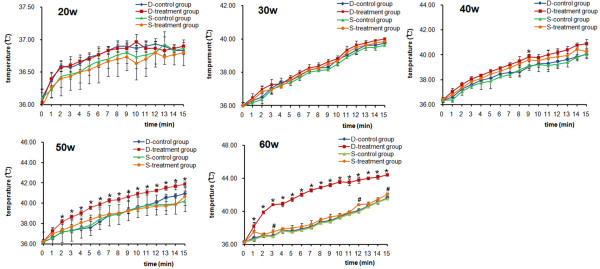
**Temperature changes in deep and superficial muscles under microwave exposure.** The sensor was inserted over the titanium alloy plate into deep muscles (5 mm above the middle hole) and superficial muscles (30 mm above the middle hole). Transient temperature variations were assessed by t-test. *P < 0.05 vs. control group in deep muscles; ^#^*P* < 0.05 vs. control group in superficial muscles. D-control group: deep muscles control group; D-treatment group: deep muscles treatment group; S-control group: superficial muscles control group; S- treatment group: superficial muscles treatment group.

Temporal temperature changes of deep muscles are shown in Figure 2 for control group and treatment group respectively. Significant differences in temperature occurred during microwave treatment using 50 w and 60 w between control and treatment groups. The temperature gaps (T_peak_-T_vally_) in deep muscles for the treatment group were significant higher compared with control group under 50w and 60 w microwave treatment (*P* = 0.037, *P* < 0.01, respectively) (Table 1). Although the deep muscle temperature increases with microwave treatment using 20 w, 30 w and 40w were higher compared with control group, no statistically significant difference was seen between the two groups. We recorded significant peak temperatures of 44.40 ± 0.17°C in the treatment group and 41.63 ± 0.21°C in the control group under 60w treatment (*P* < 0.01).

**Table 1:** **Muscle temperature variation (T**_**peak**_**-T**_**vally**_**) during microwave treatment**

**Groups**		**Temperature gaps (°C) (mean ± SD)**		***P*** **value**
		**Control group**	**Treatment group**	
**20 w**	Deep muscles	0.83 ± 0.12	0.93 ± 0.10	1.00
	Superficial muscles	0.83 ± 0.17	0.83 ± 0.21	1.00
**30 w**	Deep muscles	3.80 ± 0.30	4.00 ± 0.21	0.27
	Superficial muscles	3.67 ± 0.21	3.87 ± 0.29	0.78
**40 w**	Deep muscles	3.70 ± 0.31	4.50 ± 0.21	0.07
	Superficial muscles	3.70 ± 0.50	4.03 ± 0.55	0.17
**50 w**	Deep muscles	4.73 ± 0.20	5.63 ± 0.35	0.037*
	Superficial muscles	4.00 ± 0.81	4.40 ± 0.24	0.93
**60 w**	Deep muscles	5.33 ± 0.21	8.10 ± 0.36	<0.01*
	Superficial muscles	5.23 ± 0.21	5.80 ± 0.17	0.013*

*Differences between control and treatment group were assessed by t-test.

### Temporal temperature changes in superficial muscles

The temperature increased gradually in superficial muscles with increasing microwave dose. Although the peak temperatures for both control and treatment group were lower than 42°C, the differences between the two groups were observed under 60w treatment (*P* = 0.035) (Figure 2). In terms of temperature gaps in superficial muscles, no significant difference was detected between the two groups except for a significant temperature gap with 60w treatment (Table 1) (*P* = 0.013). No statistical difference was observed between the two groups under treatment of 20w, 30w and 40w.

## Discussion

Clinically, low-dose microwave treatment is administered to patients suffering from acute soft tissue injuries or postoperative pain [7,40]. Previous studies in vivo and in vitro suggested that a low dose of microwaves reduced edema [2,41]. It also reduced pain by facilitating the 'pain gate' mechanism [8,12]. However, whether the metal implants in areas of radiation raised the tissue temperature abnormally is still unclear. In this study, using an animal model we estimated temperature increases around metallic objects implanted, in vivo. The results suggested that no significant temperature increase occurred in the muscle tissues adjacent to the titanium alloy implants under low-dose (20 w-40 w) microwave irradiation with a 2450 MHz microwave generator. However, 50 w and 60 w caused a dramatic temperature increase in the deep muscles and even raised the temperature in the superficial muscles.

The reflection of electromagnetic waves from metal surfaces was thought to be one of the mechanisms associated with such temperature rise [42]. The implant may couple with the RF source, and thus induce a current on the implant surface. The induced current further produced a secondary electromagnetic (EM) field, with the implant acting as a weak radiating antenna in tissues [28]. Excessive dose of RF irradiation would heat the tissues adjacent to the metal implants. Theoretically, RF heating is caused by the Joule loss resulting from the sudden increase of electrical resistance at the interface between tissues and metal [43]. Compared with osseous tissue, muscle tissues contain high water content, which absorbs microwaves strongly [44]. Therefore, the present study analyzed the temperature changes of muscles adjacent to the metal implants. Additionally, the eddy current stimulated by electromagnetic field is the other reason. The physical properties of the materials contribute to the difference of eddy current intensity. Compared with traditional medical stainless steel, titanium alloy has low magnetic permeability and electrical conductivity. RF heating of cobalt-chromium alloy implants and titanium alloy implants was evaluated in vitro by Muranaka. The maximum temperature rise of titanium implant was lower compared with cobalt-chromium [33,39]. Our results indicate that, under lower dose microwave irradiation (20 w-40 w), the temperature increase of muscles adjacent to titanium alloy implants was lower than 43°C. In the previous study, enzyme and proteins degenerated at tissue temperatures over 43°C [45,46]. Radiation with the 2450 MHz microwave generator used in our study was safe for the muscle tissues adjacent to the titanium implants. However, 60w microwave exposure led to a surge of temperature to 44.5°C in deep muscle within 15 min. In clinical practice, hyperthermia is safe if the temperature was maintained below 45°C [47-49]. Significant fibrosis was reported previously only at temperatures of 46°C and above [36]. Giombini suggested that both time and temperature of application must be controlled to avoid the irreversible damage. In clinical practice, specifically in musculoskeletal medicine, temperature should not exceed 45°C for 30 min [7]. Therefore, we believe that the use of a 60w microwave therapy is unsafe for tissues implanted with titanium alloy.

High local temperature in the tissues is caused by the geometry of an implant [37,38]. In the case of implants with thin or sharp edges, electromagnetic field lines are focused along the edge or an end, leading to a high local flux [33], resulting in significant increase in absorption and temperature in tissues. In this study, no significant difference in temperature rise was observed between the ends and the middle part of the implants, due to the small size and good thermal conductivity of the implant.

Our study has a number of limitations. First, mechanical errors in one thermometer affected accuracy of temperatures recorded. The accuracy might be improved with a secondary thermometer. Second, the implants used in rabbit were the internal fixation plates of metacarpals in human. However, except for the adjacent tissues and signal frequency, other factors such as implant size, orientation, and location affect the SAR distribution and temperature increase near the implants. Therefore, experiments with larger animals should be performed. Third, in our study, the long-term effects of microwave radiation on muscle, skin and bone were not observed. We applied only a single dose of microwave diathermy. Clinical diathermy over 7–10 days on patients without metal implants relieved pain and chronic inflammation. Studies in the future should study the long-term effects of such intervention.

## Conclusion

A lower dose (20w-40w) of continuous-wave microwave irradiation delivered by a 2450 MHz microwave generator did not cause temperature increase in muscle tissue adjacent to a titanium alloy implant. However, at a dose of 50w and 60w, significant temperature increases were observed in superficial and deep muscles. Our results suggest that a low dose of microwave treatment might be a promising method for patients with titanium alloy internal fixation.

## Abbreviations

RF: Radiofrequency; EM: Electromagnetic; SAR: Specific absorption rate.

## Competing interests

The authors declare that they have no competing interests.

## Authors’ contributions

Conceived and designed the experiments: YB DY YX. Performed the experiments: DY YX TF FX. Analyzed the data: DY YX HZ WG. Contributed reagents/materials/analysis tools: DY HZ LJ. Wrote the paper: DY YB. All authors read and approved the final manuscript.

## Pre-publication history

The pre-publication history for this paper can be accessed here:

http://www.biomedcentral.com/1471-2474/14/364/prepub

## References

[B1] Dewhirst MW Viglianti BL Lora-Michiels M Hanson M Hoopes PJ Basic principles of thermal dosimetry and thermal thresholds for tissue damage from hyperthermia Int J Hyperthermia 2003 14 267 294 10.1080/0265673031000119006 12745972

[B2] Wyper DJ McNiven DR The effect of microwave therapy upon muscle blood flow in man Br J Sports Med 1976 14 19 21 10.1136/bjsm.10.1.19 963368 PMC1859362

[B3] Sekins KM Lehmann JF Esselman P Dundore D Emery AF deLateur BJ Nelp WB Local muscle blood flow and temperature responses to 915 MHz diathermy as simultaneously measured and numerically predicted Arch Phys Med Rehabil 1984 14 1 7 6691788

[B4] Yatvin MB The influence of membrane lipid composition and procaine on hyperthermic death of cells Int J Radiat Biol Relat Stud Phys Chem Med 1977 14 513 521 10.1080/09553007714551301 338522

[B5] Lehmann JF Guy AW Stonebridge JB Warren CG DeLateur BJ Temperature distribution produced in models by three microwave applicators at 433.92 megahertz Arch Phys Med Rehabil 1975 14 145 151 1119923

[B6] DeLateur BJ Stonebridge JB Lehmann JF Fibrous muscular contractures: treatment with a new direct contact microwave applicator operating at 915 MHz Arch Phys Med Rehabil 1978 14 488 499 718413

[B7] Giombini A Giovannini V Di Cesare A Pacetti P Ichinoseki-Sekine N Shiraishi M Naito H Maffulli N Hyperthermia induced by microwave diathermy in the management of muscle and tendon injuries Br Med Bull 2007 14 379 396 10.1093/bmb/ldm020 17942453

[B8] Goats GC Microwave diathermy Br J Sports Med 1990 14 212 218 10.1136/bjsm.24.4.212 2097017 PMC1478902

[B9] Chang WH Sun JS Chang SP Lin JC Study of thermal effects of ultrasound stimulation on fracture healing Bioelectromagnetics 2002 14 256 263 10.1002/bem.10009 11948604

[B10] Leon S Asbell S Arastu H Edelstein G Packel A Sheehan S Daskai I Guttmann G Santos I Effects of hyperthermia on bone. II. Heating of bone in vivo and stimulation of bone growth Int J Hyperthermia 1993 14 77 87 10.3109/02656739309061480 8433028

[B11] Leon S Asbell S Edelstein G Arastu H Daskal I Sheehan S Plunkett D Guttmann G Packel A Leon O Effects of hyperthermia on bone. I. Heating rate patterns induced by microwave irradiation in bone and muscle phantoms Int J Hyperthermia 1993 14 69 75 10.3109/02656739309061479 8433027

[B12] Lubner MG Brace CL Hinshaw JL Lee FT Jr Microwave tumor ablation: mechanism of action, clinical results, and devices J Vasc Interv Radiol 2010 14 S192 S203 10.1016/j.jvir.2010.04.007 20656229 PMC3065977

[B13] Pope G Mockett S Wright J A survey of electrotherapeutic modalities: ownership and use in the NHS in England Physiotherapy 1995 14 82 91 10.1016/S0031-9406(05)67050-2

[B14] Rabini A Piazzini DB Tancredi G Foti C Milano G Ronconi G Specchia A Ferrara PE Maggi L Amabile E Deep heating therapy via microwave diathermy relieves pain and improves physical function in patients with knee osteoarthritis: a double-blind randomized clinical trial Eur J Phys Rehabil Med 2012 14 549 559 22820824

[B15] Giombini A Di Cesare A Safran MR Ciatti R Maffulli N Short-term effectiveness of hyperthermia for supraspinatus tendinopathy in athletes a short-term randomized controlled study Am J Sports Med 2006 14 1247 1253 10.1177/0363546506287827 16636345

[B16] Giombini A Casciello G Di Cesare M Di Cesare A Dragoni S Sorrenti D A controlled study on the effects of hyperthermia at 434 MHz and conventional ultrasound upon muscle injuries in sport J Sports Med Phys Fitness 2001 14 521 527 11687773

[B17] Lehmann JF DeLateur BJ Stonebridge JB Selective muscle heating by shortwave diathermy with a helical coil Arch Phys Med Rehabil 1969 14 117 123 5774006

[B18] Schwan HP PIERSOL GM The absorption of electromagnetic energy in body tissues. A review and critical analysis Am J Phys Med Rehabil 1954 14 371 404 13207342

[B19] Sekins KM Dundore D Emery AF Lehmann JF McGrath PW Nelp WB Muscle blood flow changes in response to 915 MHz diathermy with surface cooling as measured by Xe133 clearance Arch Phys Med Rehabil 1980 14 105 113 6989343

[B20] Lehmann JF DeLateur BJ Stonebridge JB Heating patterns produced in humans by 433.92 MHz round field applicator and 915 MHz contact applicator Arch Phys Med Rehabil 1975 14 442 448 1190998

[B21] Lehmann J Dundore D Esselman P Nelp W Microwave diathermy: effects on experimental muscle hematoma resolution Arch Phys Med Rehabil 1983 14 127 6830422

[B22] Guy AW Lehmann J Stonebridge J Sorensen C Development of a 915-MHz direct-contact applicator for therapeutic heating of tissues Microwave Theory and Techniques, IEEE Transactions on 1978 14 550 556 10.1109/TMTT.1978.1129437

[B23] Kitchen SS Partridge CJ A review of microwave diathermy Physiotherapy 1991 14 647 652 10.1016/S0031-9406(10)60401-4

[B24] Grant EH Biological effects of microwaves and radio waves IEE Proceedings A Physical Science, Measurement and Instrumentation, Management and Education - Reviews 1981 14 602 10.1049/ip-a-1.1981.0091

[B25] Shields N Gormley J O'Hare N Short-wave diathermy: current clinical and safety practices Physiother Res Int 2002 14 191 202 10.1002/pri.259 12528575

[B26] Guy AW Dosimetry associated with exposure to non-ionizing radiation: very low frequency to microwaves Health Phys 1987 14 569 584 10.1097/00004032-198712000-00001 3679822

[B27] Ruggera PS Witters DM Altzahn GVM Bassen HI In vitro assessment of tissue heating near metallic medical implants by exposure to pulsed radio frequency diathermy Phys Med Biol 2003 14 2919 10.1088/0031-9155/48/17/312 14516109

[B28] Virtanen H Keshvari J Lappalainen R The effect of authentic metallic implants on the SAR distribution of the head exposed to 900, 1800 and 2450 MHz dipole near field Phys Med Biol 2007 14 1221 1236 10.1088/0031-9155/52/5/001 17301450

[B29] Cooper J Hombach V Increase in specific absorption rate in human heads arising from implantations Electron Lett 1996 14 2217 2219 10.1049/el:19961507

[B30] Yue Z Zhou J Wang X Gui Z Li L Preparation and magnetic properties of titanium-substituted LiZn ferrites via a sol–gel auto-combustion process J Eur Ceram Soc 2003 14 189 193 10.1016/S0955-2219(02)00082-1

[B31] Lee MJ Kim S Lee SA Song HT Huh YM Kim DH Han SH Suh JS Overcoming artifacts from metallic ortho-pedic implants at high-field-strength MR imaging and multi-detector CT RadioGraphics 2007 14 791 803 10.1148/rg.273065087 17495293

[B32] Paulus JA Richardson JS Tucker RD Park JB Evaluation of inductively heated ferromagnetic alloy implants for therapeutic interstitial hyperthermia Biomed Eng 1996 14 406 413 10.1109/10.486260 8626189

[B33] Muranaka H Horiguchi T Ueda Y Tanki N Evaluation of RF heating due to various implants during MR procedures Magn Reson Med Sci 2011 14 11 19 10.2463/mrms.10.11 21441723

[B34] Seiger C Draper DO Use of pulsed shortwave diathermy and joint mobilization to increase ankle range of motion in the presence of surgical implanted metal: a case series J Orthop Sports Phys Ther 2006 14 669 677 10.2519/jospt.2006.2198 17017272

[B35] Draper DO Castel JC Castel D Low-watt pulsed shortwave diathermy and metal-plate fixation of the elbow Athl Ther Today 2004 14 28 32

[B36] Goldstein L Dewhirst M Repacholi M Kheifets L Summary, conclusions and recommendations: adverse temperature levels in the human body Int J Hyperthermia 2003 14 373 384 10.1080/0265673031000090701 12745976

[B37] McIntosh RL Anderson V McKenzie RJ A numerical evaluation of SAR distribution and temperature changes around a metallic plate in the head of a RF exposed worker Bioelectromagnetics 2005 14 377 388 10.1002/bem.20112 15924346

[B38] Virtanen H Huttunen J Toropainen A Lappalainen R Interaction of mobile phones with superficial passive metallic implants Phys Med Biol 2005 14 2689 2700 10.1088/0031-9155/50/11/017 15901963

[B39] Muranaka H Horiguchi T Ueda Y Usui S Tanki N Nakamura O Evaluation of RF heating on hip joint implant in phantom during MRI examinations Nihon Hoshasen Gijutsu Gakkai zasshi 2010 14 725 733 10.6009/jjrt.66.725 20702992

[B40] Campbell R Campbell D Imaging the post-operative wrist and hand Imaging of the Hand and Wrist 2013 Springer 365 386

[B41] Ye D Xu Y Zhang H Fu T Jiang L Bai Y Effects of Low-dose microwave on healing of fractures with titanium alloy internal fixation: an experimental study in a rabbit model PloS One 2013 14 e75756 10.1371/journal.pone.0075756 24086626 PMC3784417

[B42] Skonieczki BD Wells C Wasser EJ Dupuy DE Radiofrequency and microwave tumor ablation in patients with implanted cardiac devices: is it safe? Eur J Radiol 2011 14 343 346 10.1016/j.ejrad.2010.04.004 20434862

[B43] Geczi G Horvath M Kaszab T Alemany GG No major differences found between the effects of microwave-based and conventional heat treatment methods on two different liquid foods PloS One 2013 14 e53720 10.1371/journal.pone.0053720 23341982 PMC3547058

[B44] Akyol Y Ulus Y Durmus D Canturk F Bilgici A Kuru O Bek Y Effectiveness of microwave diathermy on pain, functional capacity, muscle strength, quality of life, and depression in patients with subacromial impingement syndrome: a randomized placebo-controlled clinical study Rheumatol Int 2012 14 3007 3016 10.1007/s00296-011-2097-2 21898066

[B45] Harris ED Jr McCroskery PA The influence of temperature and fibril stability on degradation of cartilage collagen by rheumatoid synovial collagenase N Engl J Med 1974 14 1 6 10.1056/NEJM197401032900101 4357162

[B46] Yarmolenko PS Moon EJ Landon C Manzoor A Hochman DW Viglianti BL Dewhirst MW Thresholds for thermal damage to normal tissues: an update Int J Hyperthermia 2011 14 320 343 10.3109/02656736.2010.534527 21591897 PMC3609720

[B47] Guy AW Lehmann JF Stonebridge JB Therapeutic applications of electromagnetic power Proceedings Of The IEEE 1974 14 55 75

[B48] Sekins K Emery A Lehmann J MacDougall J Determination of perfusion field during local hyperthermia with the aid of finite element thermal models J Biomech Eng 1982 14 272 10.1115/1.3138359 7154647

[B49] Meshorer A Prionas SD Fajardo LF Meyer JL Hahn GM Martinez AA The effects of hyperthermia on normal mesenchymal tissues. Application of a histologic grading system Arch Pathol Lab Med 1983 14 328 334 6687797

